# A slow and silent return: a case of renal cell carcinoma recurring as gastrointestinal bleeding and small bowel obstruction

**DOI:** 10.1093/jscr/rjag834

**Published:** 2026-09-22

**Authors:** Lan Nguyen, Eric Weiss, Matthew Hudson

**Affiliations:** Department of Internal Medicine, University at Buffalo, 462 Grider St, Buffalo, NY 14215, United States; Department of Pathology and Anatomical Sciences, University at Buffalo, 100 High Street Buffalo, NY 14203, United States; Department of Medicine, Division of Gastroenterology, Hepatology, and Nutrition, University at Buffalo, 100 High Street Buffalo, NY 14203, United States

**Keywords:** renal cell carcinoma, gastrointestinal bleeding, GI bleeding, small bowel obstruction

## Abstract

Renal cell carcinoma (RCC) can metastasize years after treatment, with small bowel involvement being exceedingly rare. We report a 74-year-old man with RCC treated with nephrectomy and immunotherapy who presented ten years after clinical and radiologic remission with manifestations of metastatic disease in the small bowel. Specifically, he developed intussusception from a lead point lesion, followed by subsequent gastrointestinal (GI) bleeding and small bowel obstruction from another synchronous lesion. Each presentation required a distinct diagnostic and therapeutic approach. This case highlights the unpredictable course of RCC, underscoring the importance of comprehensive evaluation when patients with prior RCC develop new GI symptoms, regardless of time since treatment and historical remission.

## Introduction

Renal cell carcinoma (RCC) remains a common malignancy with an insidious course and the potential for late metastasis years after initial nephrectomy and chemotherapy. Small bowel metastases are rare and may present with symptoms of gastrointestinal (GI) bleeding, intussusception, or bowel obstruction. We describe a patient with a history of RCC who experienced multiple manifestations of metastatic RCC to the small bowel, highlighting the importance of a comprehensive evaluation in RCC patients presenting with new GI symptoms.

## Case report

**Figure 1:**
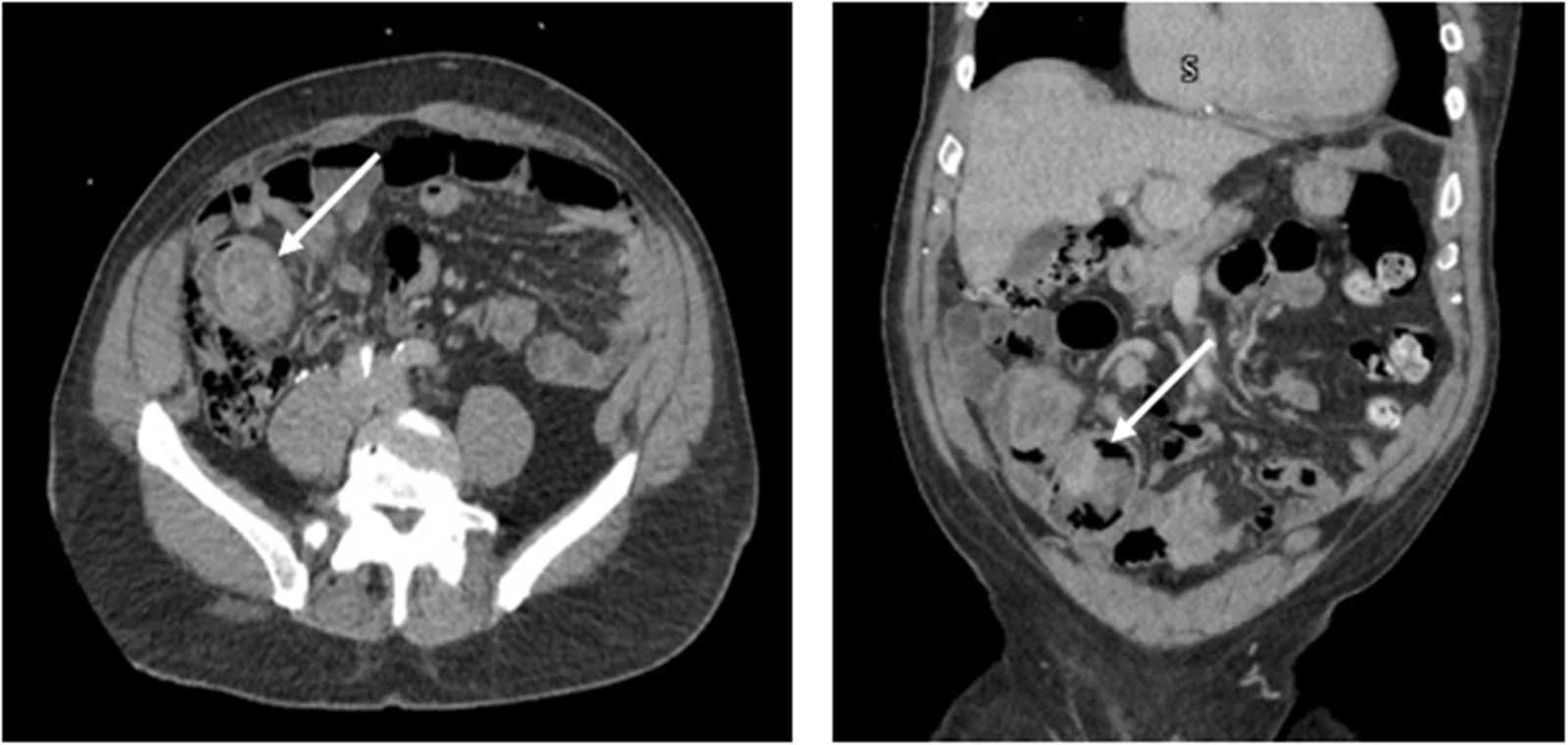
CT AP: Axial and coronal views showed a distal ileo-ileal intussusception (arrows) with associated wall thickening and hyperenhancement.

A 74-year-old male with a history of RCC treated with nephrectomy and immunotherapy presented with intermittent sharp abdominal pain. After his nephrectomy in 2014, the patient developed disease progression with an enhancing lesion inferior to the nephrectomy bed and underwent multiple immunotherapy treatments in 2015. Since 2016, he had remained off treatment and under surveillance with no evidence of disease recurrence. On presentation, the patient was hemodynamically stable but found to have a hemoglobin level of 6.7 g/dL, requiring blood transfusion. A computed tomography of the abdomen and pelvis (CT AP) showed an 8-cm distal ileo-ileal intussusception with wall thickening and hyperenhancement (Fig. 1). He subsequently underwent a small bowel resection, with pathology confirming a lead point RCC metastasis as the site of intussusception.

**Figure 2:**
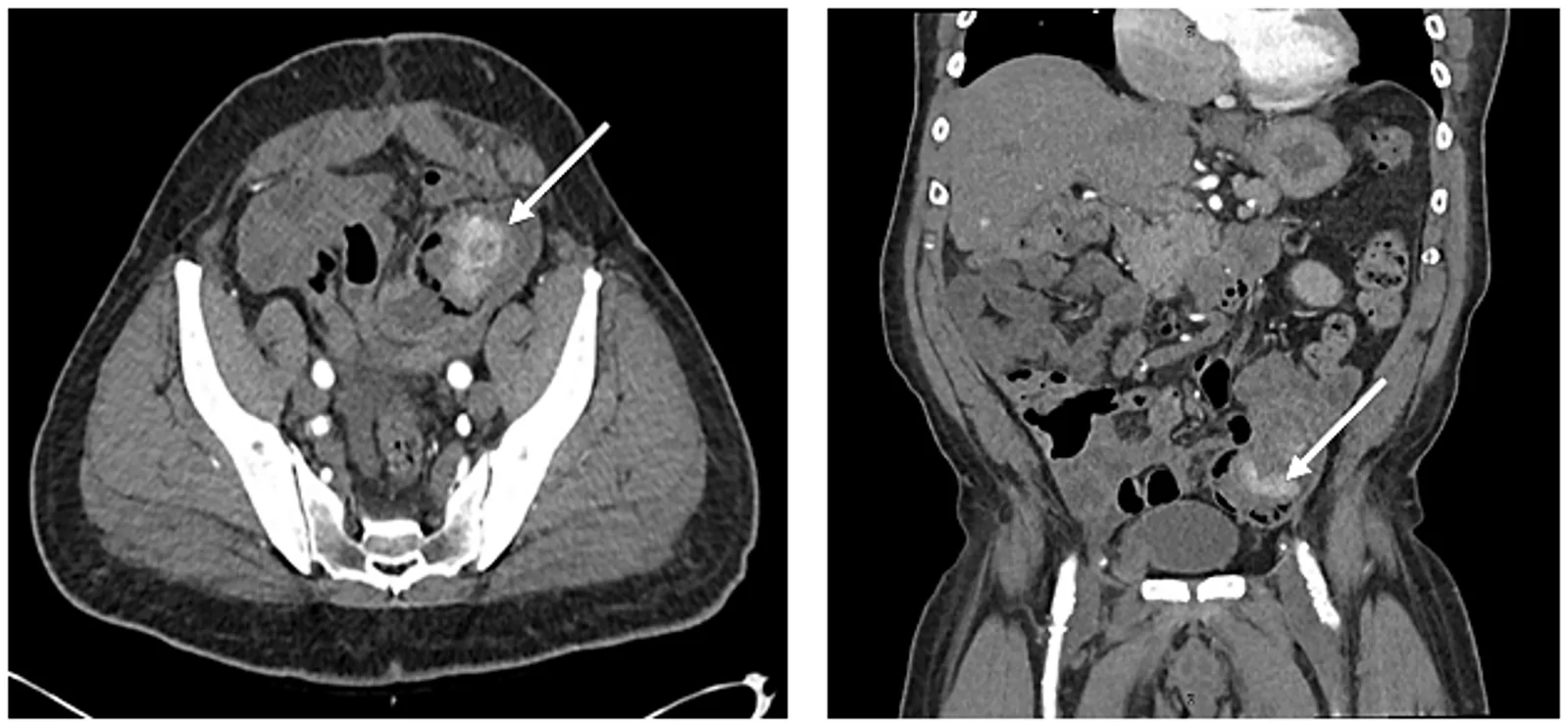
CTA abdomen and pelvis: Axial and coronal views showed an area of active extravasation of contrast in the left lower quadrant (arrows).

**Figure 3:**
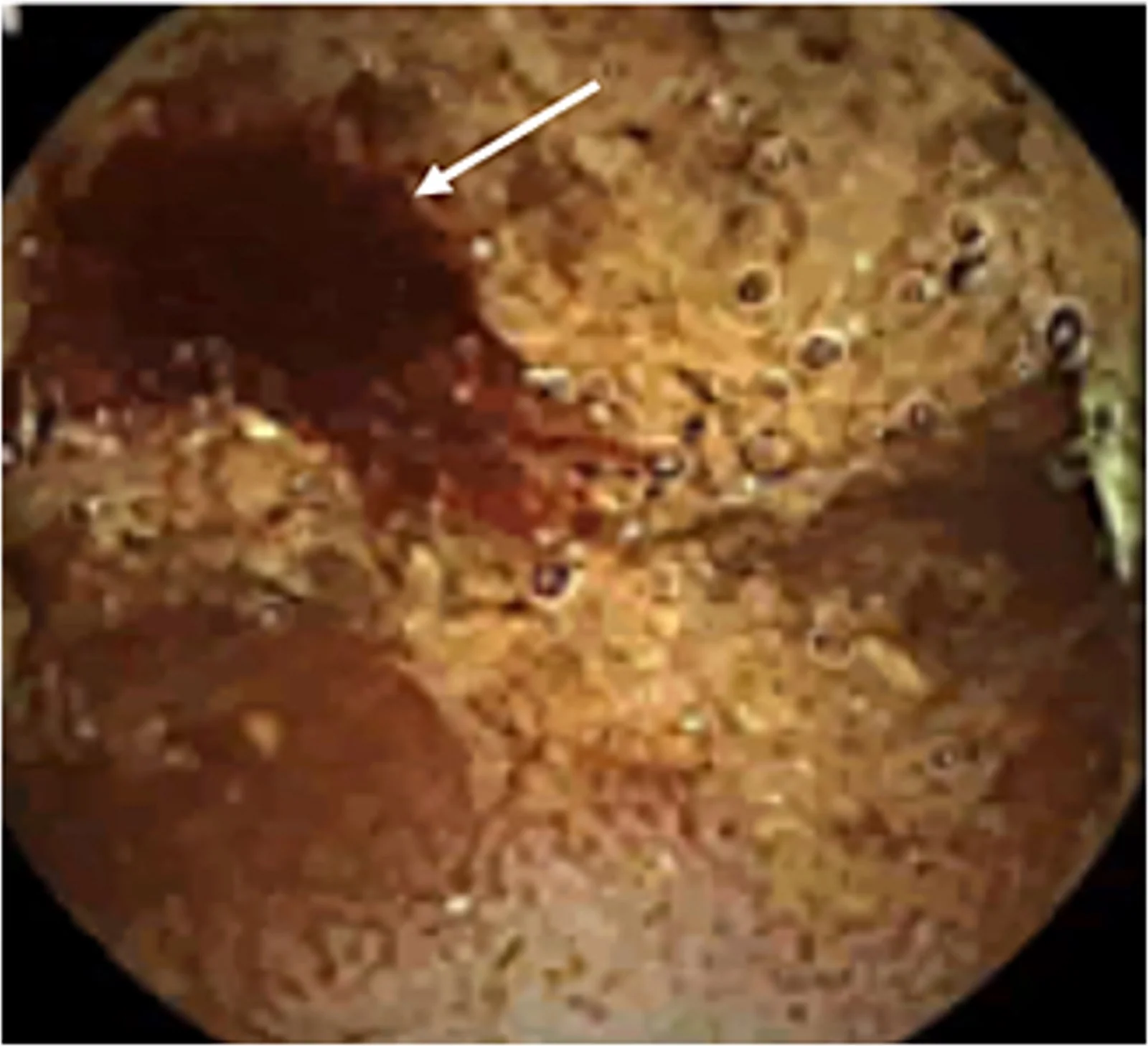
Video capsule endoscopy identified a blood clot in the ileum (arrow).

Three weeks later, he returned with one week of intermittent hematochezia. On arrival, his vital signs and physical examination were unremarkable. Laboratory studies showed a hemoglobin level of 5.4 g/dL (previous postoperative baseline was 8–10 g/dL), prompting blood transfusions. Esophagogastroduodenoscopy and colonoscopy within the prior year had not demonstrated pathologies explaining his hematochezia. Computed tomography angiography (CTA) of the abdomen and pelvis revealed active extravasation within a loop of small bowel, arising from a distal branch of the superior mesenteric artery (Fig. 2). Given his recent surgery, normal prior endoscopy, and the absence of balloon-assisted enteroscopy at our facility, further endoscopic procedures were deferred in favor of a video capsule endoscopy (VCE). VCE confirmed recent blood in the distal small bowel, aligning with the bleeding site observed on CTA. The source of bleeding, however, was obscured by clot and debris (Fig. 3). A referral for balloon-assisted enteroscopy or laparoscopically assisted enteroscopy was recommended but declined by the patient given resolution of ongoing blood loss.

**Figure 4:**
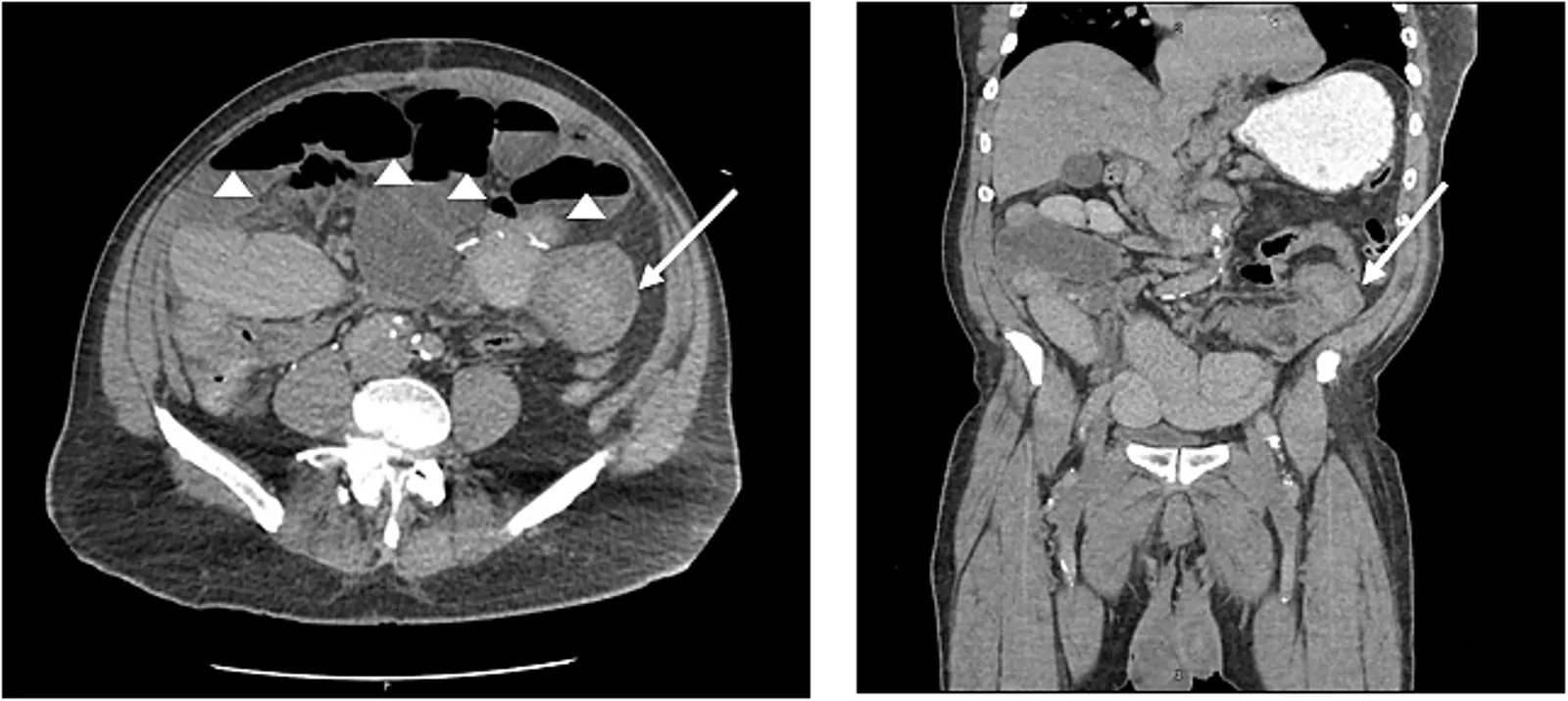
CT AP with oral and intravenous contrast: Axial view showed small bowel dilation with multiple air-fluid levels (arrowheads) indicating a small bowel obstruction. Axial and coronal view revealed and intussusception in the left lower quadrant (arrows).

**Figure 5:**
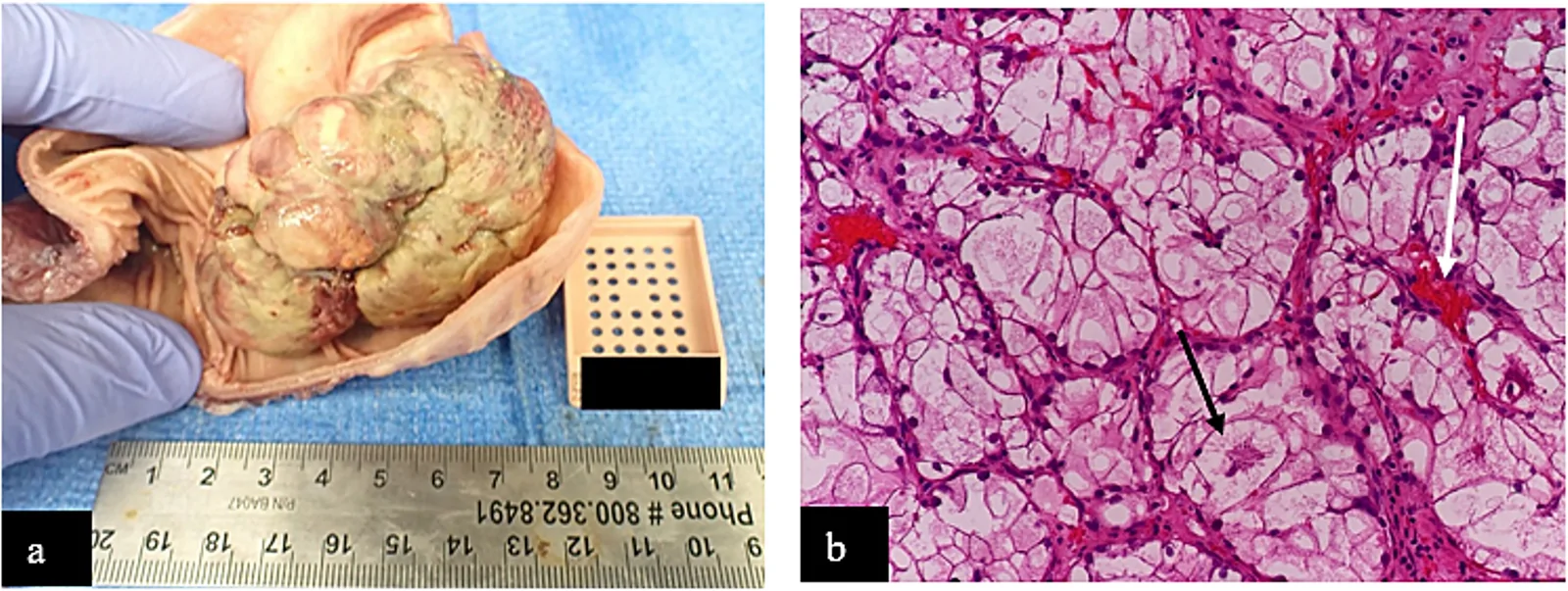
(a) An obstructing mass measuring 7.5 × 5×3 cm located in the distal small bowel. (b) Classic histological findings of renal clear cell carcinoma, including clear cytoplasm with distinct cell boundaries (black arrow) and network of thin-walled, ‘chicken-wire’ vasculature (white arrow). Magnification 400×.

Three weeks later, he presented with two days of abdominal distention and vomiting. He was hemodynamically stable, and laboratory results were unremarkable. However, physical examination revealed generalized abdominal tenderness. Repeat CT AP showed a high-grade small bowel obstruction due to recurrent intussusception near the surgical anastomosis, with evidence of intraluminal hemorrhage (Fig. 4). A nasogastric tube was placed for decompression, followed by an emergent surgical exploration. Another lead-point mass causing intussusception was identified intraoperatively, and the patient underwent another small bowel resection. Pathology again confirmed metastatic RCC with clear margins (Fig. 5). Postoperatively, he had significant symptom relief and was discharged with oncology follow-up for further treatment options of recurrent metastatic RCC.

## Discussion

Accounting for 90% of solid renal tumors, RCC is characterized by an indolent clinical course [1]. At the time of diagnosis, ~30% of patients have metastatic disease [1]. RCC most commonly metastasizes to the lungs (50%–60%), bones (30%–40%), liver (30%–40%), and brain (5%) [2–5]. Small bowel metastases are exceedingly rare, with an incidence of just 0.7% [6]. When present, the duodenum is the most frequent site, particularly in right-sided RCC due to its tendency for locoregional spread [7]. In contrast, jejunal and ileal metastases, as seen in this patient, are less common and often associated with vascular invasion by the tumor [8]. Metastatic disease may occur years after initial treatment. In this case, multifocal ileal involvement was identified ten years after nephrectomy and eight years following response to immunotherapy. While Fujii *et al*. revealed that only 10% of patients develop metastatic disease beyond five years post-nephrectomy [9], another case series by Ritchie and deKernion reported that the large majority occur later [10]. Given this variability, clinicians should maintain a high level of suspicion for small bowel metastasis in RCC patients who present with new GI symptoms, regardless of the time since initial treatment.

The most common manifestation of small bowel metastasis from RCC is GI bleeding, resulting from direct tumor invasion, vascular involvement, or vascular nature of RCC [8]. Other symptoms, including bowel obstruction, intussusception, or perforation result from mass effect of the tumor. For patients without active GI bleeding, computed tomography with intravenous contrast can detect bowel wall edema, obstruction, or a lead point mass [11]. In cases of active GI bleeding, CTA is valuable for identifying contrast extravasation and hence, localizing the bleeding site [12]. Although VCE is beneficial when bleeding is suspected to originate beyond the reach of traditional enteroscopy, it does not allow for tissue sampling. Meanwhile, balloon-assisted enteroscopy offers both visualization and biopsy, making it a key tool for diagnosing RCC metastases to the small bowel [13].

Treatments are influenced by the patient’s overall health and disease burden. Surgical resection is preferred for isolated metastatic lesions. A study by Alt *et al*. revealed that RCC patients who underwent resection had a longer survival rate (4.8 years) compared to those who did not undergo surgery (1.3 years) [14]. For patients who are not surgical candidates but have a good or intermediate prognosis, targeted therapies are effective options [8]. For cases of active GI bleeding due to metastatic RCC, angiographic embolization of tumor-supplying arteries is often required, although this carries a risk of bowel ischemia and lacks long-term data on outcomes [15]. For patients with widespread metastatic disease, treatments should prioritize palliative approaches aimed at symptom relief and improving quality of life.

## Conclusion

Small bowel metastases from RCC are rare and can manifest with various GI symptoms including bleeding, intussusception, or intestinal obstruction. In addition to this, the unpredictable nature of RCC underscores the importance of a thorough evaluation of new GI symptoms in patients with a history of RCC to facilitate timely diagnosis, guide management, and improve patient outcomes.

## Prior presentation

This case was previously presented at the Digestive Disease Week, took place on 5/5/2025 in San Diego, California.
